# Knowledge, attitude and practice of staff of 4 hospitals in Yaoundé on the prevention of vertical transmission of hepatitis B

**DOI:** 10.11604/pamj.2017.28.174.10971

**Published:** 2017-10-25

**Authors:** Talla Paul, Tebeu Pierre Marie, Efuetnkeng Bechem

**Affiliations:** 1 Yaoundé General Hospital, Yaoundé, Cameroon; 2 Yaoundé University Teaching Hospital and Faculty of Medicine and Biomedical Sciences, University of Yaoundé 1, Yaoundé, Cameroon; 3 Bamenda Regional Hospital, Yaoundé, Cameroon

**Keywords:** PMTCT/HBV, hepatitis B, healthcare providers, knowledges, attitudes and practices

## Abstract

**Introduction:**

Hepatitis B virus infection is a public health concern in Cameroon and worldwide. With hepatitis C virus, it is the first cause of liver cancer in Cameroon. The high prevalence of 11.9% in Cameroon is associated with the premature contamination at the perinatal period, due to vertical transmission, from mother-to-child. To put into practice the preventives measures, actors need a good knowledge on premature contamination of a baby. The general objective of this study was to evaluate the influence of level of knowledge on the attitudes and the professional practices concerning prevention of mother-to-child transmission of hepatitis B (PMTCT/HBV) in Yaoundéhospitals and environs.

**Methods:**

We carried out a cross sectional multicentric, KAP study from 10^th^ March to 15^th^ December 2015 in the obstetrics services of 4 hospitals in Yaoundéand environs. For each health care provider who gave his consent, we used a pretested questionnaire to collect socio-demographics and professional data as well as their knowledges, attitudes and practices on PMTCT/HBV. After given a grade to each item, we proceeded to a quantitative analysis of data using SPSS software and Epi info 7^th^ version.

**Results:**

105 health care provider took part in the study, made up of 82 women (79%) and 22 men (21%). The ages were between 23 and 60 years, with a mean age of 40.9 ± 9.2 years. Only 21% of the participants had good knowledges on HBV/PMCT. This knowledge had a significant link with the profession, the professional experience and the duration in the same service. All the nurseaids had inadequate knowledges as well as the elders in the profession. Most of the participants (64.4%) had favorableattitude on PMTCT/HBV and that was significantly associated to good knowledges. (OR:5.34; CI 95% [1.47-19.47], p = 0.006). The practices on PMTCT/HBV were inappropriate in 57.1% of the participants. There were no significant relation between good knowledge and the practices (OR: 1.818, CI 95% [0.705-4.68]; p = 0.213) as well as between good attitudes and practices on PMTCT/HBV (OR: 0.932; CI 95% [0.423-2.058]; p = 0.862).

**Conclusion:**

The healthcare provider in hospitals in Yaoundé and its environs are old. Their knowledge on PMTCT/HBV is inadequate and their practices inappropriate. Good knowledge doesn't always lead to good practices of PMTCT/HBV. There exist some obstacles or intermediate variables between good knowledge, good attitudes and appropriate practices of PMTCT/HBV.

## Introduction

Hepatitis B is an inflammatory process of the liver caused by a viral infection that is found worldwide. The World Health Organisation (WHO) estimated that in 2015 about 240 million people were chronic carriers of hepatitis B virus (HBV) [1]. The severity of this affection is by its possible progression to late complications like liver cirrhosis and primitive liver cancer. HBV is a communicable disease whose mode of transmissions are well known; by blood and blood products, sexual transmission and vertical transmission from mother-to-child. Vertical transmission has been identified to be one of the causes of the high prevalence of HBV infection in Sub-Sahara Africa [2, 3]. Actually, an early contamination in the perinatal period will provoke a chronic infection in 90% of infected children. This progressive risk is only 5% if the contamination occurs in adult life [4-6]. Therefore prevention of mother-to-child transmission (PMTCT) of HBV (PMTCT/HBV) constitute one of the main pillars in the fight against this infection in regions of high prevalence like Cameroon [7-11]. The main actors in the implementation of this prevention are health care providers in charge of pregnant women whose daily action should contribute to reduce the mother-to-child transmission of HBV, as is the case of HIV/AIDS. In Cameroon this strategy of prevention is still embryonic. The PMTCT of HBV varies with hospitals and even amongst practitioners.

**Objectives**: This study is to evaluate the knowledge, attitude and practice of health staff ofhospitals in Yaoundé and its environs (Cameroon), on PMTCT/HBV.

## Methods

**Type, place, period of study**: We carried out a cross sectional and analytical KAP study. Four health facilities in Yaoundé and its environs were selected for the study essentially on the basis of the number of deliveries per annum which varied from 800 to 3500 for hospitals in Yaoundé. The study took place from the 1^st^ March 2015 to the 31^st^ December 2015 after approbation by the Cameroon National Ethical Committee.

**Sampling**: The sampling was consecutive and exhaustive, with voluntary recruitment. Were included in the study all healthcare providers involved in the management of pregnant women in the health facilities. Non-included were health care providers on internship and administrative staff. Those who were eligible but did participate in the study were either absent (permission, annual leave, sick leave, maternity leave) or did not give their consent. We assessed the knowledge, attitude and practice of healthcare providers in the maternity on PMTCT/HBV and the level of influence of the knowledge on their attitude and practice on PMTCT/HBV.

**Variables**: Data were collected using a pre-tested questionnaire with the presence of an interviewer. The questionnaire had 4 sections: a section on socio-demographic and professional data, a section on knowledge on PMTCT/HBV (epidemiology, natural history and complications), a section on attitude following risk of MTCT/HBV and a section on practice of PMTCT/HBV by health care providers. Data on knowledge, attitude and practice on PMTCT/HBV were graded with maximum grades of respectively 10, 6 and 10. Knowledge on PMTCT/HBV of participants were grade from 1 to 10 and was quoted “good” if the grade was greater of equal to 7 and “insufficient” if less than 7. Assessment of attitude of care givers took into consideration their beliefs and perception of risk of MTCT/HBV and answers were graded from 1 to 6. A grade above 4 was quoted “favourable” or “good” attitude and a grade less than or equal to 4 was quoted “poor” attitude. Professional practice was grade from 1 to 10. It was quoted “poor or “inadequate” if the grade was less than 7 and “good” if the grade was greater than or equal to 7.

**Statistical analysis**: Data were analysed using SPSS 18 of IBM corporation. Chi square test and odds ratio were used accordingly and level of significance was p < 0.05.

## Results

Amongst the 139 participant in the study, 105 gave their consent representing a participation rate of 75.54%. 68.6% of participants were from the Yaoundé General Hospital and Yaoundé Gynaeco-obstetric and Paediatric Hospital, while only 9.5% were from Mfou District Hospital (Table 1). The ages of the participants ranged from 23 to 60 years with an average age of 40.9 ± 9.2 years. 79% (83 of 105) of respondent were female. Health care providers from the maternity were recruited in the study; amongst who were nurse aids, assistant midwives, state registered nurses, general practitioners, residents in obstetrics/gynaecology and obstetricians/gynaecologists (Table 2). 45.7% of the participants had a professional experience from 11 to 20 years, while only 11.4% had an experience of less than 2 years (Table 2). Amongst the 105 participants, 61 (58.1%) were Catholics against 7 (6.7%) Muslims (Table 2). The average grade of knowledge of participants on PMTCT/HBV was 5.5 ± 1. Amongst the participants 83 (79%) had “inadequate” knowledge on PMTCT/HBV (score < 7/10); only 22 participants (21%) had “good” knowledge (7-10/10). The level of knowledge was assessed to be insufficient in all age groups, especially in the more than 50 years where only 5.9% had good knowledge on PMTCT/HBV, but was similar in both sex. The level of knowledge varied with the professional hierarchy. No nurse aid had a good knowledge on PMTCT/HBV, while 52.6% of doctors had good knowledge with a statistical significant difference (p = 0.001) (Table 3). All participants in the study with a working experience of more than 20 years had insufficient knowledge (Table 3). The level of knowledge was inversely proportionate to the working experience (47.1% of good knowledge for less than 1 year experience against 7.4% for working experience of more than 10 years, p = 0.31) (Table 3). When we assessed the attitude of health care provider on PMTCT/HBV, 64 (61%) had a “good” attitude. Analysis of the influence of knowledge on attitude of health care providers revealed a close association between knowledge and professional attitude. 86.4% (19 out of 22) of participant having good knowledge on PMTCT/HBV also had good professional attitude, against 54.2% (45 out of 83) of participants having insufficient knowledge. Having a good knowledge on PMTCT/HBV increased the chances of good professional practice by 5.35 times. This association is important and statistically significant; p = 0.006; OR: 5.34; (95% CI: (1.47-19.47)) (Table 4). The average score of participants on professional practice on PMTCT/HBV was 6.4 ± 1.9. 45 participants (42.9%) had good professional practice while 60 participants (57.1%) had poor practices. Meanwhile, 12 out of 22 participants(54.5%) having good knowledge on PMTCT/HBV also had good professional practices, against 33 out of 83 participants (39.8%) who had insufficient knowledge but carried out good practices. This difference was not statistically significant (p = 0.213) (Table 5). 27 out of 64 participants (42.2%) who had good professional attitude also had good professional practices, against 18 of the 41 participants (43.9%) having a poor attitude but carried out good professional practices (p = 0.862).

**Table 1:** Distribution of participant and health facilities

Health Facility	Number of Participants N (%)
YGH	36 (34.3)
CASS Nkoldongo	23 (217.9)
Mfou DH	10 (9.5)
YGOPY	36 (34.3)

YGH: Yaoundé general hospital, CASS: DH: district hospital, YGOPY: Yaoundé gynaeco-obstetric and paediatrics hospital

**Table 2:** Socio demographic and professional characteristics of participants

Variables	Group	Number N=105 (%)
Age Group (years)	≤ 30	18 (17.1)
	31- 40	28 (26.7)
	41- 50	42 (40)
	> 50	17 (16.2)
Sex	Male	22 (21)
	Female	83 (79)
Profession	Nurse aid	11 (10.5)
	Assistant midwives	40 (38.1)
	State registered nruse	23 (21.9)
	Midwives	12 (11.4)
	General Practitioners	4 (3.8)
	Residents Obs/Gyn	9 (8.6)
	Obstetricians/Gynaecologist	6 (5.7)
Religion	Catholic	61 (58.1)
	Protestant	25 (23.8)
	Pentecostal	8 (7.6)
	Muslim	7 (6.7)
	Others	4 (3.8)
Professionnal (years) experience	< 2	12 (11.4)
	2 - 5	13 (12.4)
	6 -10	19 (18.1)
	11 - 20	48 (45.7)
	> 20	13 (12.4)
Duration in service (years)	< 2	38 (36.2)
	2 -5	20 (19.0)
	6 - 10	20 (19.0)
	> 10	

**Table 3:** Level of knowledge on PMTCT/HBV with regards to some socio-demographic and professional characteristics

Variables		Level of knowledge		TOTAL N= 105 N(%)	p
		Good Knowledge; N1= 22 N1(%)	Insufficient Knowledge N2= 83 N2 (%)		
Sex	Female	17 (77.3)	66 (79.5)	83 (79)	
	Male	5 (22.7)	17 (20.5)	22 (21)	0.818
Age Group (years)	≤ 30	6 (273)	12 (14.4)	18 (17.1)	
	31 - 40	6 (27.3)	22 (26.5)	28 (26.7)	
	41 – 50	9 (40.9)	33 (39.8)	42 (40.0)	0.261
	> 50	1 (4.5)	16 (19.3)	17 (16.2)	
Profession	Nurse Aid	0 (0)	11 (13.3)	11 (10.5)	
	Assistant Midwive	5 (22.7)	35(42.2)	40 (38.1)	
	Midwives/Nurses	7 (31.8)	28 (33.7)	35 (33.3)	
	Doctors	10 (45.5)	9 (10.8)	19 (18.1)	0.001
WorkingExperience (Years)	< 2	5 (22.7)	7 (8.4)	12 (11.4)	
	2 - 5	3 (13.6)	10 (12.0)	13 (12.4)	
	6 - 10	8 (36.4)	11 (13.3)	19 (18.1)	0.009
	11 - 20	6 (27.3)	42 (50.6)	48 (45.7)	
	> 20	0 (0)	13(15.7)	13(12.4)	
Duration in service (years)	< 1an	8 (36.4)	9 (10.8)	17 (16.2)	
	[1 - 2]	4 (18.2)	17 (20.5)	21 (20.0)	
	[2 - 5]	5 (22.7)	15 (18.1)	20 (19.0)	0.031
	[6 - 10]	3 (13.6)	17 (20.5)	20 (19.0)	
	> 10	2 (9.1)	25 (30.1)	27 (25.8)	
Religion	Catholic	11(50.0)	50 (60.2)	61(58.1)	
	Protestant	7 (31.8)	26 (31.3)	33(31.4)	0.388
	Muslim and Others	4 (18.2)	7 (8.4)	11(10.5)	
HealthFacility	YGH	10 (45.5)	26 (31.4)	36 (34.3)	
	CASS Nkoldongo	3 (13.6)	20 (24.1)	23 (21.9)	
	YGOPY	7 (31.8)	29 (34.9)	36 (34.3)	0.586
	MFOU DH	2 (9.1)	8 (9.6)	10 (9.5)	

**Table 4:** Influence of level of knowledge on attitude participants on PMTCT/HBV

		Attitudes		Odds ratio (CI 95%)	p
	Total N (%)	Good n(%)	Poor n(%)		
Knowledge Good	22 (100)	19 (86.4)	3 (13.6)	1	0.006
Insufficient	83 (100)	45 (54.2)	38 (45.8)	5. 34 [1.47 – 19.47]	

**Table 5:** Influence of level of knowledge and attitude on professional practice on PMTCT/HBV

Independent Variables		Professional practice		Odds ratio (CI 95%)	p
	Total n (%)	Good n (%)	Poor n (%)		
**Level of Knowledge** Good	22 (100)	12 (54.5)	10 (45.5)	1	0.213
Insufficient	83 (100)	33 (39.8)	50 (60.2)	1.818 [0.705-4.689]	
**Attitudes** Good	64 (100)	27 (42.2)	37 (57.8)	1	0.862
Poor	41 (100)	18 (43.9)	23 (56.1)	0.932 [0.423 – 2.058]	

## Discussion

We carried out a study on 105 participants in 4 different hospitals in Yaoundé and its environs. The ages of the participants ranged from 23 to 60 years with an average age of 40.9 ± 9.2 years. 56.2% of participants had more than 40 years. The average age of participants in our study is more than observed by Fouwou Njoya [12], on health care providers in Yaoundé which was 32.4 ± 9.3 years. This difference can be explained by the fact that in the latter study were included interns and voluntary workers who are generally younger than the permanent staff. 79% of our participants were female with a sex ratio of 0.26. This reflects a high attraction of female workers to the health section and especially reproductive health sector. This tendency is observed in Africa and beyond. In their paper published in 2003, Cognet and Fortin reported that 91% of paramedics in Quebec were female [13]. This feminine attraction can be explained by the motherly instinct of women always ready to protect human lives. When we assessed the working experience of the participants we observed that 76.2% had more than 5 years of working experience and 58.1% more than 10 years. These findings are similar to those of FouwouNjoya [12] who found 75.6% of health care givers to have a more than 5 years working experience. This similarity can be explained by the fact that in Cameroon a good number of paramedics are trained but not recruited in the public service, where these two studies were carried out. In 2015, only 40 state registered nurses where recruited into the public service in Cameroon [14]. We also noticed that 27.7% of participants had an experience of more than 10 years in the same service while 36.2% had less than 2 years experience in the same service. During our study we observed that 58.1% of participants were catholic in faith, while 23.8% were protestant and 6.7% Muslims. This can be explained by the fact that the study took place in the South of the country where Christians are majority and also by the fact that one of the hospitals where participants were recruited is a Catholic confessional hospital. We noticed in our study that 79% of the participants had insufficient knowledge in PMTCT/HBV. This result is similar to that of MOUJONGUE [15] who found that 70.59 of health care providers in the Yaoundé Teaching Hospital had insufficient knowledge on PMTCT/HBV. Meanwhile in a similar study the same year, FOUWOU NJOYA [12] observed that 57.8% of health care givers had insufficient knowledge on PMTCT/HBV. In a study carried out on 518 nurses working in perinatal services in California, USA, [16] observed that less than 25% of participants had good knowledge on viral hepatitis B. This absence of knowledge on PMTCT/HBV can be explained by several reasons; training programmes for heath personnel doesn't include extensive modules on hepatitis B virus prevention and refresher courses on PMTCT/HBV are not carried out in our hospitals. This is even truer as there isn't a national preventive programme on hepatitis B virus. In our study we did not notice any influence on gender (p = 0.818), age (p = 0.261), religion (p = 0.388), nor health facility (p = 0.586) on the level of knowledge. This can be explained by the fact that in Cameroon, there is no discrimination of sex, age, or religion on education.

We also found out that knowledge was closely related to level of professional (p = 0.001), to working experience (p = 0.009) and to working years in obstetrical services (p = 0.031). Actually services provided vary with health facilities and some minimum knowledge is provided to health personnel actors of management of pregnant women infected with hepatitis B virus. The relationship between knowledge and working experience shows that working experience is not proof of knowledge in PMTCT/HBV. This indicates that refresher courses for personnel are necessary to improve their knowledge on PMTCT/HBV. We also noticed in this study that the number of working years in the obstetrical service is not gage of good knowledge on PMTCT/HBV. This is explained by the fact that the younger generation receives a better formation in this domain as training modules are revised to adapt current disease affections. The younger generation is also more acquainted to the use of internet and uses this tool to acquire better information on some affections. We observed that 64.4% of participant had a favourable attitude on PMTCT/HBV. The assessment of this parameter took into consideration believes and the ways of behaving towards a risk of mother-to-child contamination of hepatitis B virus. There is little in the literature on this parameter. Participants having good knowledge on PMTCT/HBV also had good attitudes. This can be explained by the fact that knowledge on complications of hepatitis B virus infection drives each individual to adopt attitudes to prevent the infection. This implies that improving on the knowledge of personnel of PMTCT/HBV can increase improve their attitude by 5.34 times. We realised in our study that 57.1% of health care givers had poor professional practice on PMTCT/HBV. This finding is similar to that of FOUWOU NJOYA [12] who reported that 60% had poor professional practice on PMTCT/HBV. This can be explained by the fact that lack of qualified personnel on PMTCT/HBV, absence of refreshing on the subject and absence of national directives on PMTCT/HBV. On analysing the influence of knowledge on professional practice, we did not find a statistically significant relationship. Actually, having good knowledge on PMTCT/HBV produces good professional practice (OR: 1.82) but not statistically significant (p = 0.213). This can be explained by the fact that there exist other determinants of good professional health practices which were not analysed by this study. Likewise, analysis of attitude on professional practice indicates that there isn't any relationship between attitude and professional practice (p = 0.86). This signifies that having good attitude doesn't necessary implies good professional practice, meaning that there are obstacles preventing the implementation of good attitude in practice. These obstacles are present when the care giver has to take decisions. These obstacles are called “intermediate variables” according to NOUMBISSIE [17] in his theory of “enlarged model of planned behaviour”. Among these intermediate variables are non-availability of screening tests and other virological tests on hepatitis B virus, the cost of screening of hepatitis B virus infection, the very costly anti- hepatitis B virus immunoglobulins, the scarcity of the anti-hepatitis B immunoglobulin, absence of national directives or programmes to prevent hepatitis B infection like the one for HIV/AIDS, absence of logistics to optimise PMTCT/HBV programmes, ignorance of pregnant women on risk of maternal hepatitis B infection. Only a qualitative study can better explain these obstacles and intermediate variables.

## Conclusion

This study revealed that health care providers in obstetrical services in hospitals in Yaoundé are aging and their level of knowledge on PMTCT/HBV is insufficient. There exist a strong association between good knowledge and attitude on PMTCT/HBV. Meanwhile their professional practice is inadequate. These practices are not influenced neither by the level of their knowledge nor by their attitude. We also noticed that there are obstacles or intermediate variables between having good knowledge and carrying out good practices on PMTCT/HBV. It is therefore necessary to renew the staff of obstetrical services and to organise refresher courses to health care providers on PMTCT/HBV, and the identification of obstacles impeding the implication of knowledge on PMTCT/HBV.

### What is known about this topic

- Hepatitis B vertical transmission is known to exist in carrier mothers;
- Modes of prevention of vertical transmission have been described during antenatal consultations, labour and delivery and post-partum.

### What this study adds

- This study comes to highlight the impact and evaluate of the knowledge on hepatitis B vertical transmission in health personnel and their attitudes and practices towards this infection;
- It helps to know if acquisition of knowledge is reflected in the attitude and practices of health personnel in the prevention of vertical transmission of hepatitis B.

## Competing interests

The authors declare no competing interests.
